# The C-Type Lectin of the Aggrecan G3 Domain Activates Complement

**DOI:** 10.1371/journal.pone.0061407

**Published:** 2013-04-15

**Authors:** Camilla Melin Fürst, Matthias Mörgelin, Kasper Vadstrup, Dick Heinegård, Anders Aspberg, Anna M. Blom

**Affiliations:** 1 Department of Laboratory Medicine, Division of Medical Protein Chemistry, Lund University, Malmö, Sweden; 2 Department of Clinical Sciences, Division of Infection Medicine, Lund University, Lund, Sweden; 3 Department of Biology, University of Copenhagen, Copenhagen, Denmark; 4 Department of Clinical Sciences, Section of Rheumatology, Lund University, Lund, Sweden; Radboud University Nijmegen Medical Centre, The Netherlands

## Abstract

Excessive complement activation contributes to joint diseases such as rheumatoid arthritis and osteoarthritis during which cartilage proteins are fragmented and released into the synovial fluid. Some of these proteins and fragments activate complement, which may sustain inflammation. The G3 domain of large cartilage proteoglycan aggrecan interacts with other extracellular matrix proteins, fibulins and tenascins, via its C-type lectin domain (CLD) and has important functions in matrix organization. Fragments containing G3 domain are released during normal aggrecan turnover, but increasingly so in disease. We now show that the aggrecan CLD part of the G3 domain activates the classical and to a lesser extent the alternative pathway of complement, via binding of C1q and C3, respectively. The complement control protein (CCP) domain adjacent to the CLD showed no effect on complement initiation. The binding of C1q to G3 depended on ionic interactions and was decreased in D2267N mutant G3. However, the observed complement activation was attenuated due to binding of complement inhibitor factor H to CLD and CCP domains. This was most apparent at the level of deposition of terminal complement components. Taken together our observations indicate aggrecan CLD as one factor involved in the sustained inflammation of the joint.

## Introduction

The complement system provides defense against foreign pathogens but it also acts as a sensor of danger, aiding in the removal of dying cells, immune-complexes and misfolded molecules [1]. Misguided or excessive complement activation can on the other hand contribute to a wide range of autoimmune disorders and pathological inflammatory conditions such as rheumatoid arthritis (RA) [2]. Complement activation products can be found in synovial fluids of patients with active RA, and a role for complement in RA is supported by the protective effect of deficiencies of complement proteins in arthritis mouse models as well as therapeutic effect upon complement inhibition in these models [3].

Complement can be activated via three pathways; the classical pathway is triggered by binding of various ligands such as clustered IgG and IgM antibodies, C-reactive protein, DNA and lipopolysaccharide to the C1-complex consisting of the recognition protein C1q, and two copies each of the proteolytic subunits C1s and C1r [4]. The lectin pathway is initiated when mannose-binding lectin (MBL) or ficolins bind to specific carbohydrate structures or acetylated ligands [1] while the alternative pathway is commenced by autoactivation of the unstable complement factor C3 and its subsequent deposition on activating pathogen surfaces.

During pathologic cartilage destruction, cartilage proteins are fragmented and released into the synovial fluid where they can interact with complement. This has been proposed to contribute to the local pro-inflammatory milieu in joints of patients suffering from RA. C1q, the initiator of the classical pathway, binds to decorin [5], [6], biglycan [5], fibronectin [7], laminin [8], osteoadherin [6], fibromodulin [9], cartilage oligomeric matrix protein (COMP) [10] and more weakly to lumican [6] and chondroadherin [6]. These interactions can result in inhibition of C1q (decorin, biglycan, COMP) or in activation of the classical pathway (fibromodulin, osteoadherin). Interestingly, those extracellular matrix (ECM) molecules that activate C1q and the ensuing complement cascade also bind complement inhibitors such as factor H (FH) [6] and C4b-binding protein (C4BP) [11] in order to limit inflammation. Furthermore, COMP, an established marker of joint destruction, activates the alternative complement pathway [10], [12].

**Figure 1:**
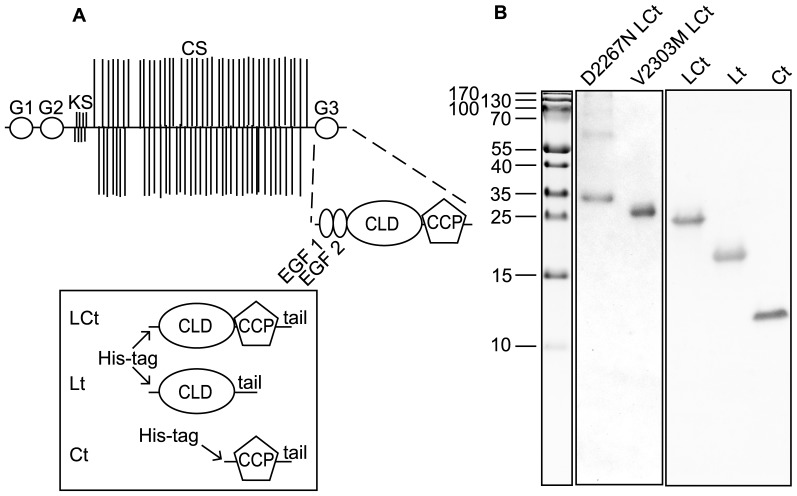
Structure of aggrecan and localization of G3 domain fragments which were used in the study. **A**, schematic outline of human aggrecan, including three globular domains (G1–G3), which is involved in numerous interactions with other ECM components. The carboxyl-terminal globular G3 domain consists of one or two EGF-like domains, a C-type lectin domain (CLD) and a complement control protein (CCP) domain followed by a short tail. In the current study we used three wild-type recombinant fragments of G3 domain: LCt, Lt and Ct and two mutants of LCt containing D2267N or V2303M substitutions. **B**, the wild-type and mutated fragments of G3 domain were expressed in eukaryotic cells and separated by SDS/PAGE followed by Coomassie Blue staining.

In the present study we investigated if aggrecan, which is the major proteoglycan in the articular cartilage, may also engage in interaction with complement. Aggrecan is expressed by chondrocytes and it is heavily substituted with chondroitin sulphate (CS) and keratan sulphate (KS) glycosaminoglycan chains, which retain water, resulting in a pressure-resistant gel structure that provides cartilage with its load-bearing properties. Aggrecan is also involved in chondroskeletal morphogenesis during development [13]. The glycosaminoglycan carrying region is flanked by globular domains that mediate binding to other ECM molecules (Fig. 1A) [14]. The N-terminal G1 domain interacts with link protein attached to hyaluronan to organize aggrecan into larger units [15]. The C-terminal G3 domain binds via the C-type lectin domain (CLD) to the ECM proteins tenascins [16], [17], fibulins [18], [19] and fibrillin [20]. The aggrecan G3 domain exists in different splice variants in man, with optional epidermal growth factor-like domains (EGF) and complement control protein (CCP) domain, which might fine-tune interactions between CLD and its ligands [17]. G3 also has a role in post-translational processing of the aggrecan core protein and the subsequent secretion [21]. Mutations localized to the CLD, V2303M and D2267N, are associated with familial osteochondritis dissecans and spondyloepimetaphyseal dysplasia, respectively [22], [23]. In the former disorder parts of articular cartilage and subchondral bone dislodge from the joint surface and early onset osteoarthritis (OA). The latter disorder is characterized by severe chondrodysplasia. The D2267N mutation introduces a novel glycosylation site whereas the V2303M mutation likely leads to conformational changes, both leading to disruption of the interactions between CLD and its ECM ligands [22], [23].

Aggrecan turnover is mediated by degradation by aggrecanases and matrix metalloproteinases. The aggrecan monomer is cleaved at several well-defined positions, and the C-terminal G3 and variable parts of the CS-rich part of the molecule are progressively liberated from cartilage into synovial fluid. The parts containing the G1 domain are retained longer in the tissue due to binding to hyaluronan [24], [25]. The cleavages by aggrecanases are thought to increase in joint diseases such as RA and OA. There are indications that aggrecan might activate complement, shown by the ability of full-length bovine aggrecan to induce the formation of soluble C5b-9 (membrane attack complex, MAC) [26]. In the present study we further dissected the complement activation properties of intact aggrecan as well as of the globular domain G3.

## Materials and Methods

### Ethics Statement

Ethical approval for the study was obtained from the ethics committee of Lund University (permit number 418/2008); written consent was obtained from all participants (healthy volunteers) who donated blood for preparation of serum. Fetal, calf and adult bovine cartilage were obtained in the form of waste from a Scan slaughterhouse in Kävlinge, Skåne, Sweden after obtaining permission from this slaughterhouse to use these animal parts. According to Swedish laws and regulations no ethical permit is required for use of such waste in research.

### Proteins and Sera

Recombinant proteins corresponding to splice variants of the G3 domain of aggrecan were expressed from the pCEP4-BM40-hisEK vector in embryonic kidney 293-EBNA cells and purified as described previously [17], [22]. The identity of the constructs was confirmed by mass spectrometry and purity verified by electrophoresis (Fig. 1B). The constructs (LCt, Lt, Ct) contain an N-terminal His-tag and combinations of a CLD (L), a CCP domain (C) and a tail (t) (Fig. 1A). Two disease-associated mutants of the LCt construct were created containing V2303M [22] and D2267N [23] substitutions. The D2267N mutation was introduced through QuickChange (Stratagene) site-directed mutagenesis using primers 5′-TGG ATC GGC CTG AAC AAC CGT ACG ATC GAA GGG GAC TTC CGC-3′ and 5′-GCG GAA GTC CCC TTC GAT CGT ACG GTT GTT CAG GCC GAT CCA-3′. The V2303M mutation was introduced according to [22]. The mutant proteins were expressed and purified as described for the wild type constructs. Purity was verified by electrophoresis and the larger mass of D2267N compared to wild type is due to a glycosylation of the substituted asparagine residue (Fig. 1B).

Intact aggrecan was extracted with 4 M guanidine-HCl from fetal, calf and adult bovine articular cartilage, and purified by CsCl-density gradient centrifugation, as described in [27]. Aggrecan used for electron microscopy was extracted and purified from bovine nasal cartilage using the same protocol.

Normal human serum (NHS) was prepared from freshly drawn blood, which was allowed to clot for 30 min at RT and 1 h on ice. Each incubation step was followed by centrifugation. The serum fractions were pooled and stored in aliquots at −80°C. To prepare heat-inactivated serum (hi-NHS), NHS was incubated at +56° for 60 min. C3, C3b and properdin were from Complement Technology. C1q [28], C4BP [29] and FH [30] were purified from plasma as described, with additional purification of FH. Samples containing FH were applied to an MRCOX-24 affinity column, unbound proteins were washed away with 1 M NaCl in TBS and FH was eluted with 3 M MgCl_2_ (pH 7.0) followed by immediate dialysis against TBS.

### Deposition of Complement Proteins from Serum and Depleted Sera

Microtiter plates (Maxisorp, Nunc) were coated with 5 µg/ml LCt, V2303M LCt, D2267N LCt (untreated or deglycosylated by N-glycosidase F (29 U/mg, O/N at 37C; Roche Applied Science), Lt and Ct recombinant fragments of aggrecan G3 domain in 75 mM sodium carbonate buffer (pH 9.6), O/N at 4°C. Ligands known to activate the respective pathways of complement were coated and used as positive controls; aggregated human IgG (5 µg/ml, classical pathway; Immuno), mannan (50 µg/ml, lectin pathway; M-7504 Sigma), acetylated BSA (10 µg/ml, lectin pathway; Applichem, acetylated as described in [31]) or zymosan (30 µg/ml, alternative pathway; Z-4250, Sigma). BSA does not activate human complement and 1% BSA (Applichem) in PBS was used as negative control. Plates were blocked with 1% BSA in PBS for 2 h at RT. Increasing amounts of NHS, C1q-depleted serum (Quidel) or factor B (FB)-depleted serum (Complement Technology) in 50 mM Hepes pH 7.35, 100 mM NaCl, 0.15 mM CaCl_2_, 1 mM MgCl_2_ (classical and lectin pathway) or Mg^++^EGTA (2 mM veronal buffer, pH 7.4, 60 mM NaCl, 120 mM glucose, 0.1% gelatin, 10 mM EGTA, 7 mM MgCl_2_; alternative pathway) were added. Deposited complement components were detected with specific antibodies, after incubation at 37°C for 20 min for C3b and C4b (A063 and Q0369, Dako) deposition, or 45 min for C1q (A0136, Dako), C9 (A226, Complement Technologies), MBL (HYB 131-10, Antibodyshop), ficolin 2 [32] and ficolin 3 [33] (kindly provided by P. Garred, University of Copenhagen, Copenhagen, Denmark) deposition. For alternative pathway activation, the incubation was for 35 min. The secondary antibodies were HRP-conjugated (Dako) and plates were developed with *o*-phenylenediamine substrate (Dako) and H_2_O_2_ and the absorbance at 490 nm was measured using Cary 50 MPR microplate reader, Varian.

Whole aggrecan (10 µg/ml) preparations extracted from fetal, calf and adult bovine articular cartilage were coated on 96-well Flexible PVC Microplates (BD Falcon) in 75 mM sodium carbonate buffer (pH 9.6), O/N at 4°C. C4b deposition through the classical pathway was determined as described above, with the exception that the incubation with NHS was shortened to 10 min.

### Direct Binding Assays

To assess the binding of C1q, FH, C4BP, C3 and properdin to aggrecan, Maxisorp plates were coated with 5 µg/ml LCt, V2303M LCt, D2267N LCt, Lt and Ct and appropriate positive controls; aggregated IgG (5 µg/ml, C1q-binding), recombinant bovine fibromodulin (2.5 µg/ml, FH- or C4BP-binding), properdin (5 µg/ml, C3-binding) or C3 (10 µg/ml, properdin-binding) in 75 mM sodium carbonate buffer (pH 9.6), O/N at 4°C. Negative control was 1% BSA in PBS. Wells were blocked with 1% BSA for 2 h at RT. C1q, FH, C4BP, C3 and properdin or hi-NHS were diluted in binding buffer (50 mM Hepes pH 7.4, 150 mM NaCl, 2 mM CaCl_2_, 50 µg/ml BSA) and added to wells at increasing concentrations. Binding of properdin was also assessed from NHS, NHS supplemented with 10 mM EDTA to block complement activation and FB-depleted serum. After incubation for 1 h at 37°C (C1q) or at RT (FH, C4BP, properdin) or O/N at RT (C3), bound proteins were detected with specific antibodies against C1q, FH (A312, Quidel), C4BP (PK9008, in house), C3b and properdin (A239, Complement Technology), followed by HRP-conjugated secondary antibodies (Dako).

To determine the effect of ionic strength, Ca^2+^ and other divalent ions on C1q and FH binding to aggrecan constructs, plates were coated and blocked as previously. Purified C1q (12 µg/ml) and FH (50 µg/ml) were diluted in binding buffer supplemented with increasing concentrations of NaCl (150–600 mM) or in 50 mM Hepes pH 7.4, 150 mM NaCl, 5 mM EDTA, 50 µg/ml BSA. Binding of C1q and FH was detected after 1 h incubation at 37°C or at RT, respectively, as previously described.

### Electron Microscopy

The localization of C1q molecules bound to aggrecan was analyzed by negative staining and transmission electron microscopy as described previously [34]. C1q was mixed with intact, fully glycosylated bovine nasal cartilage aggrecan and the anti-aggrecan-gold conjugates and incubated for 30 min at 4°C. Five microliter aliquots containing 10 nM protein were adsorbed onto carbon-coated grids for 1 min, washed with two drops of water, and stained with two drops of 0.75% uranyl formate. The grids were rendered hydrophilic by glow discharge at low pressure in air. Electron microscopy was done at the Core Facility for Integrated Microscopy (CFIM), University of Copenhagen, with a CM 100 BioTWIN electron microscope operated at 80 kV accelerating tension. Images were acquired with an OSIS Veleta digital camera.

## Results

### Aggrecan G3 Domain Activates Classical but not Lectin Pathway

**Figure 2:**
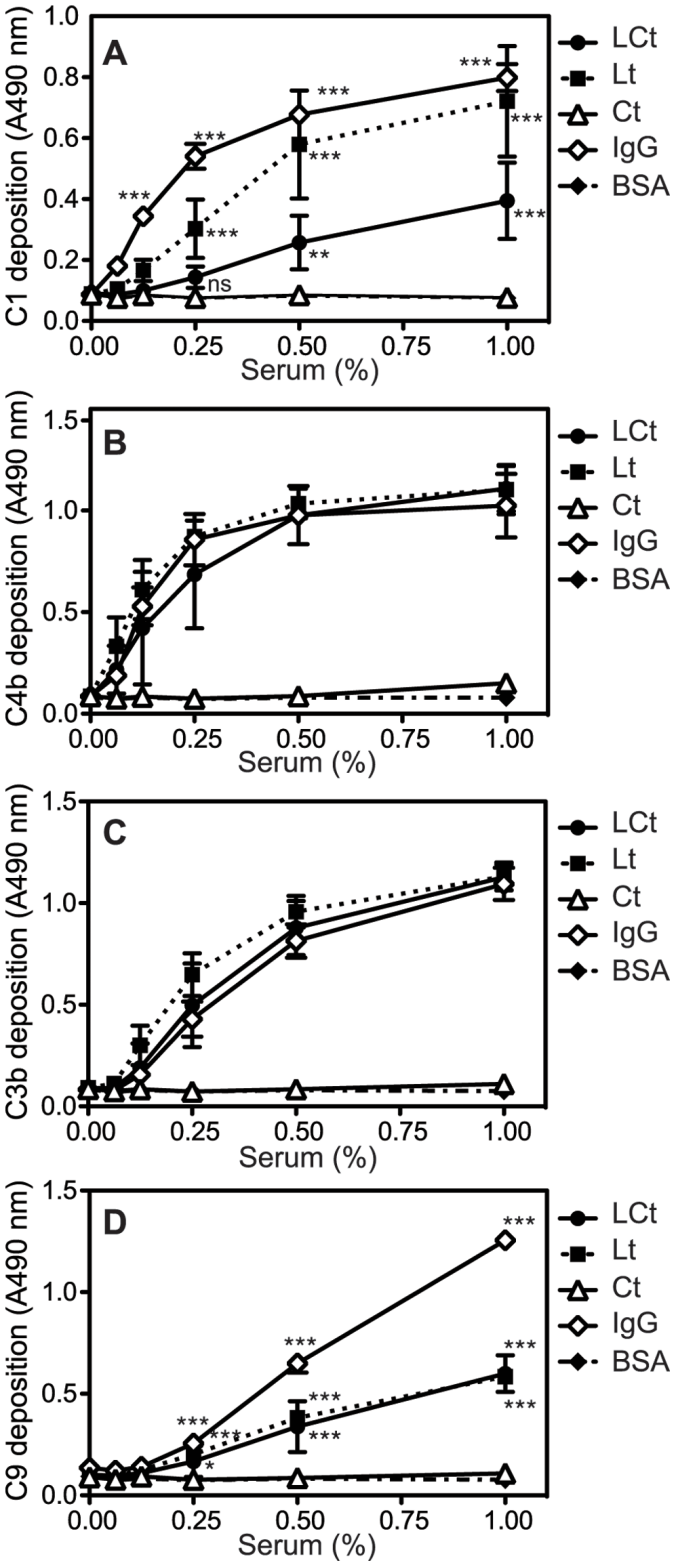
Lectin domain of aggrecan G3 module activates the classical pathway. Aggregated IgG (positive control for classical pathway), LCt, Lt, Ct and BSA (negative control) were coated onto microtiter plates and increasing concentrations of normal human serum (NHS) were added. Activation of the classical pathway was measured by detecting deposited C1q (**A**), C4b (**B**), C3b (**C**) and C9 (**D**). The graphs show the mean and standard deviation (SD) of three independent experiments. Statistical significance of differences was calculated using a two-way ANOVA with a Bonferroni posttest. * p<0.05, **p<0.01, ***p<0.001.).

The purity of the G3 domain constructs used in this study (Fig. 1B) was verified by electrophoresis on a 15% Tricine gel. We then assessed the ability of fragments of aggrecan G3 domain to activate complement by coating proteins onto microtiter plate wells followed by incubation with increasing concentrations of NHS and detection of deposited complement proteins with specific antibodies. C-type lectin-containing fragments (LCt and Lt) induced strong deposition of C4b and C3b to a similar extent as for positive control IgG (Fig. 2B and C), whereas the Ct fragment lacking the lectin domain did not. Moreover, C1 was deposited strongly on Lt and to a lower degree also on LCt, which indicates complement activation through the classical pathway (Fig. 2A). Less of the MAC component C9 was detected on LCt and Lt compared to IgG but this deposition was still significantly higher than background observed for BSA (Fig. 2D). Similarly, less deposited C5 was detected on LCt and Lt compared to IgG (not shown).

**Figure 3:**
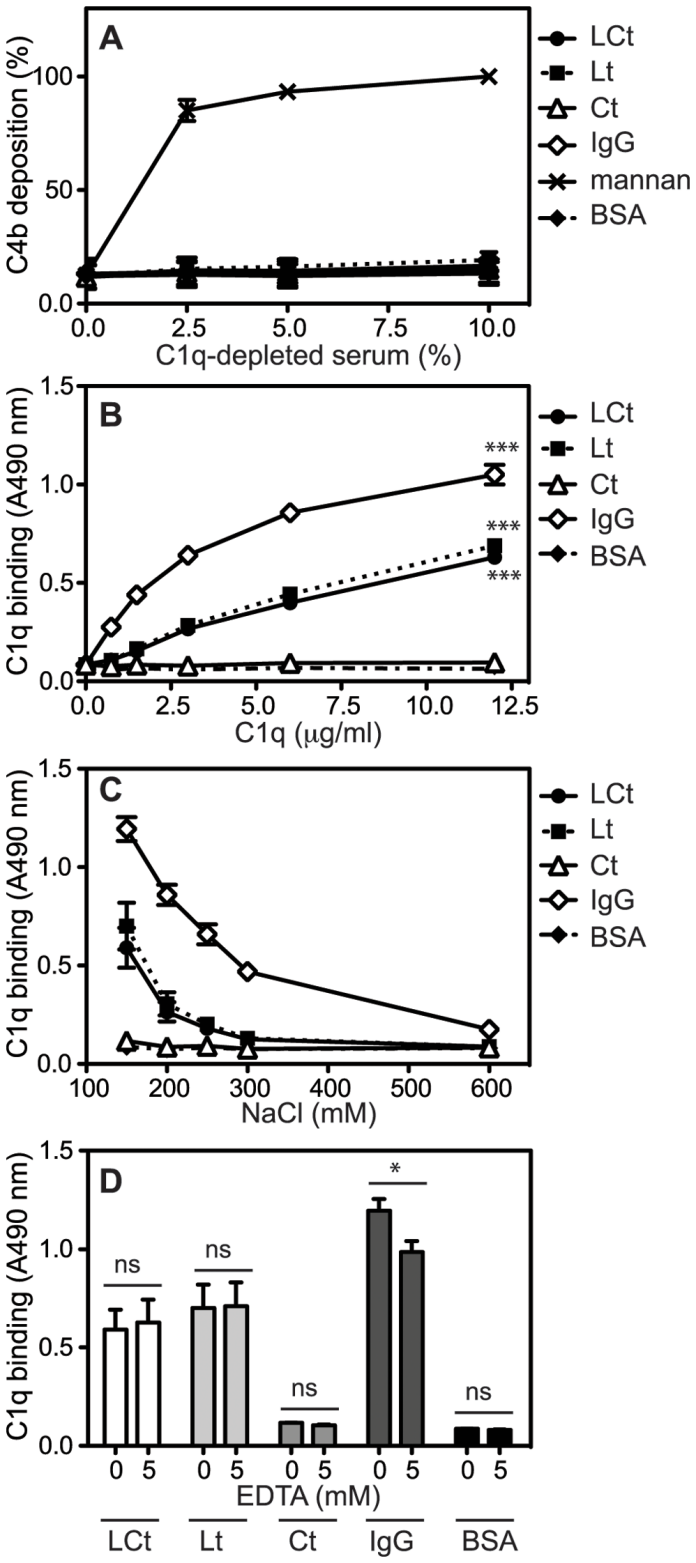
Lectin domain of aggrecan G3 domain activates classical pathway due to binding of C1q. **A**, aggregated IgG (positive control for classical pathway), mannan (positive control for lectin pathway), LCt, Lt, Ct and BSA (negative control) were coated onto microtiter plates and increasing concentrations of C1q-depleted human serum were added. Activation of the classical pathway was measured by detecting deposited C4b. The absorbance was normalized relative to deposition on mannan from 10% C1q-depleted serum. **B**, aggregated IgG, LCt, Lt, Ct and BSA were coated onto microtiter plates and incubated with purified C1q, which was then detected with specific antibodies. Aggregated IgG, LCt, Lt, Ct and BSA were coated onto microtiter plates and incubated with purified C1q in a buffer supplemented with increasing NaCl concentrations (**C**) or EDTA (**D**). The graphs show the mean and SD of three independent experiments. Statistical significance of differences was calculated using two-way ANOVA with Bonferroni posttest (B, D). (*p<0.05, **p<0.01, ***p<0.001.).

Further, none of the lectin pathway activators such as MBL, ficolin 2 or ficolin 3 were deposited on the G3 fragments, demonstrating the absence of lectin pathway activation (not shown). We also assessed deposition of C4b from C1q-depleted serum, in order to confirm that classical but not lectin pathway is responsible for the observed activation in Fig. 2 B–C. The deposition of C4b on IgG, LCt and Lt fragments was abrogated using C1q-depleted serum, while deposition on mannan (positive control for lectin pathway) was maintained (Fig. 3A). Similar was observed when testing deposition of C3b (not shown).

### Lectin-containing G3 Fragments Bind C1q

Using a microtiter-based assay, purified C1q was shown to bind LCt and Lt fragments as well as IgG (Fig. 3B). The binding of C1q to LCt/Lt fragments was dependent on ionic interactions, and was completely disrupted by 300 mM NaCl (Fig. 3C). The presence of EDTA that chelates calcium ions, did not affect the interaction between C1q and LCt/Lt fragments (Fig. 3D), while the binding of C1q to IgG was slightly affected. Further, an additional recombinant aggrecan G3 variant, corresponding to the longest splice form, encompassing the LCt construct with two additional N-terminal EGF domains [17], was confirmed to bind C1q to the same extent as LCt (not shown). This indicates that the C1q binding site is still available even when the EGF domains are present.

### C1q Binds to the Aggrecan G3 Domain and to Additional Sites on the Aggrecan Core Protein

**Figure 4:**
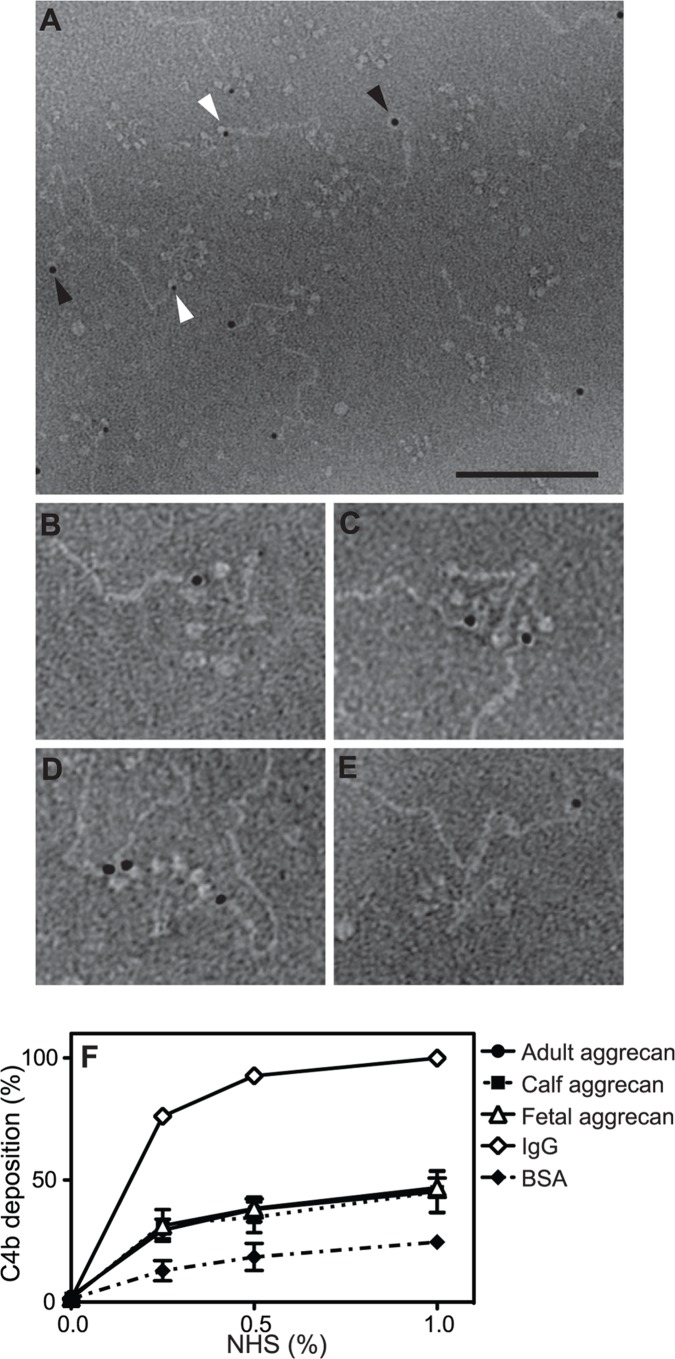
C1q binds the G3 domain and a part of the aggrecan core protein. **A–E,** using electron microscopy, complexes between bovine nasal cartilage aggrecan and C1q were visualized by negative staining. G1 and G3 domains of aggrecan were labelled with antibody-conjugated colloidal gold (10 nm and 5 nm, respectively) and are pointed out by black (G1) and white (G3) arrowheads. Scale bar shows 100 nm (A) and 50 nm (B). **A**, several aggrecan molecules bound to the heads of C1q through their G3 domains. C1q also bound the core of aggrecan. **B–D**, selected complexes between single molecules of C1q and one to three aggrecan molecules through their G3 domains, at a higher magnification. **E**, single molecule of C1q bound to the core of aggrecan. **F**, microtiter plates were coated with aggregated IgG (positive control), adult, calf and fetal bovine articular cartilage aggrecan and BSA (negative control) and deposited C4b from increasing concentrations of NHS was detected. The graph show the mean and SD of three independent experiments. Statistical significance of differences was calculated using two-way ANOVA with Bonferroni posttest. (*p<0.05, **p<0.01, ***p<0.001.).

To further verify the G3 domain as the C1q binding site, we used electron microscopy to study the complexes between gold-labelled full-length aggrecan and C1q. The head regions of C1q appear to bind most frequently (79%) to the G3 domains pointed out by antibodies conjugated with 5 nm colloidal gold (Fig. 4A–D). Interactions mediated by the heads of C1q are typical for other complement-activating ECM proteins [6]. Interestingly, C1q bound in 21% of cases also to the aggrecan core protein in the KS- or CS-attachment region (Fig. 4A and E) indicating presence of additional binding sites.

### Aggrecan from Bovine Cartilage of Different Ages Activates Complement

In order to confirm that also whole, intact aggrecan has capacity to activate complement, aggrecan was extracted from fetal, calf or adult bovine articular cartilage. Whole aggrecan activated the classical pathway of complement, as shown by C4b deposition (Fig. 4F) and there was no significant difference in activation with age of animal from which aggrecan was isolated.

### A Disease-associated Mutation in the C-type Lectin Domain within the G3 Domain Decreases the Complement Activation Capacity

**Figure 5:**
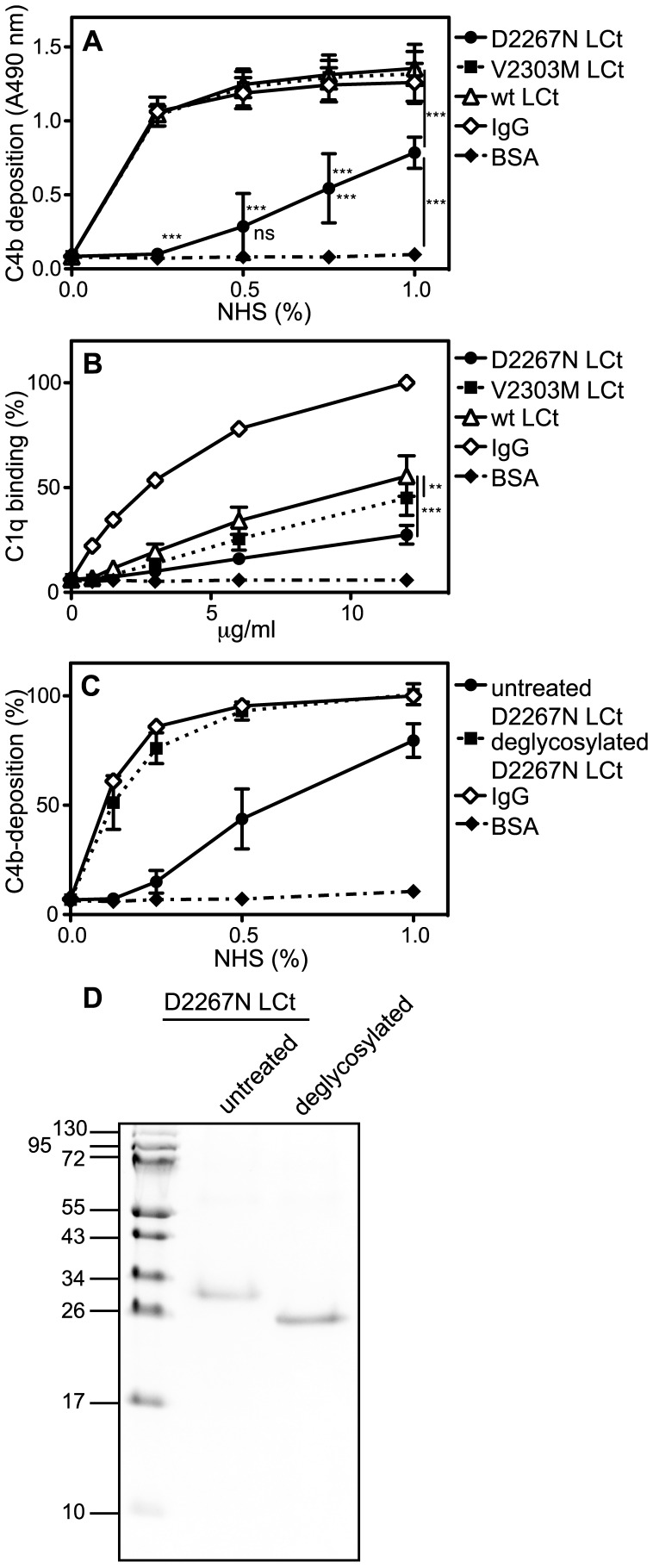
The mutation D2267N within the G3 domain decreases the complement activation capacity. **A–B**, microtiter plates were coated with aggregated IgG (positive control for classical pathway), LCt carrying the mutation D2267N or V2303M, wt LCt, and BSA (negative control). **A**, increasing concentrations of NHS were added and complement activation was measured by detecting deposited C4b. **B**, plates were incubated with purified C1q, which was then detected with specific antibodies. **C**, Microtiter plates were coated with untreated or N-glycosidase F-treated D2267N LCt, aggregated IgG and BSA. Proteins were incubated with increasing concentrations of NHS and deposited C4b was detected. Note that in addition to deglycosylation, the deamidation resulting from N-glycosidase F treatment reverts the protein sequence to wild type. **D**, D2267N LCt was deglycosylated with N-glycosidase F and untreated and deglycosylated proteins were separated by SDS/PAGE followed by Coomassie Blue staining. The graphs show the mean and SD of three independent experiments. Statistical significance of differences was calculated using two-way ANOVA with Bonferroni posttest. (*p<0.05, **p<0.01, ***p<0.001.).

We further evaluated whether two different disease-associated mutations positioned in the G3 domain would affect the complement activating properties. The mutations V2303M and D2267N are associated with osteochondritis dissecans and spondyloepimetaphyseal dysplasia, respectively. The LCt construct containing the V2303M mutation maintained its capacity to activate complement through the classical pathway, while the D2267N mutant lost some of its capacity compared to wild type (Fig. 5A). The binding of C1q to the D2267N mutant was also decreased in comparison to wild type (Fig. 5B). The D2267 residue coordinates one of the calcium residues needed to stabilize the loops forming the binding surface of the ECM ligands [35]. However, since the complement factor interactions occur in the presence of EDTA, it appears likely that effect of the D2267N mutation is related to the novel N-linked glycosylation site formed. Alternatively, the overall fold of the CLD could be disturbed by the mutation. To exclude gross misfolding, we treated D2267N LCt with N-glycosidase F, which resulted in an increased capacity of the protein to activate complement compared to untreated mutant protein (Fig. 5C) and removal of the glycosylation (Fig. 5D). However, since N-deglycosylation with this enzyme results in the replacement of the glycan-carrying asparagine residue with aspartate (i.e. in this case a reversion to wild-type sequence), we cannot conclusively say that the glycosylation per se causes the decreased complement activation.

### Aggrecan G3 Domain Weakly Activates Initial Stages of the Alternative Pathway

**Figure 6:**
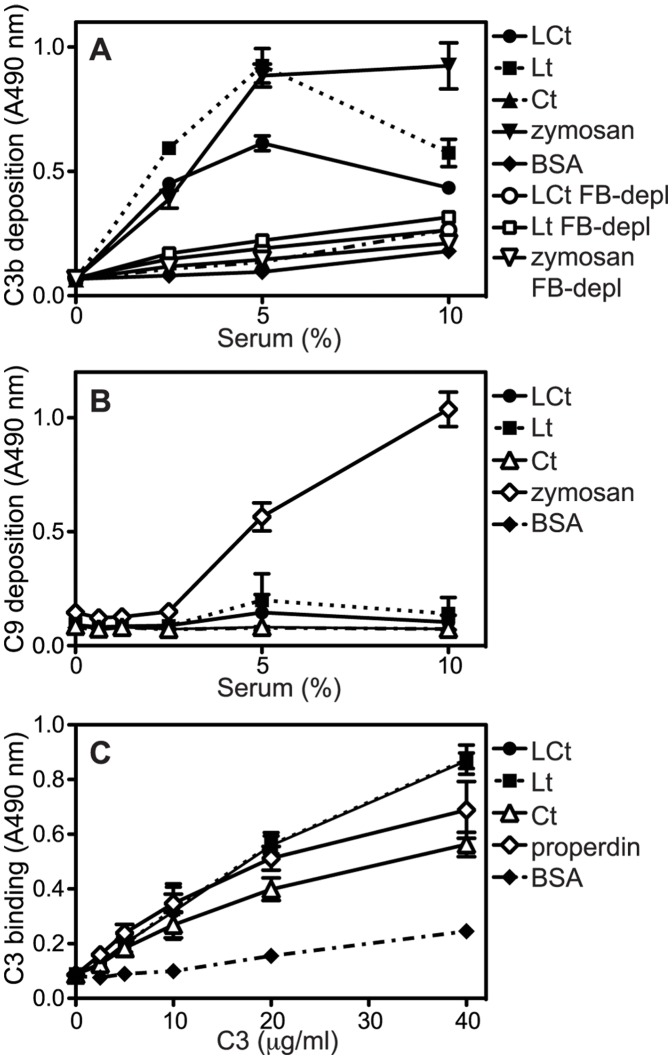
Lectin domain of aggrecan G3 module triggers limited activation of the alternative pathway. Zymosan (positive control for alternative pathway), LCt, Lt, Ct and BSA (negative control) were coated onto microtiter plates and increasing concentrations of NHS or factor B-depleted (FB-depl) human serum in Mg++EGTA were added. Activation of the alternative pathway was measured by detecting deposited C3b (**A**) and C9 (**B**). Properdin (positive control for binding of C3), LCt, Lt, Ct and BSA (negative control) were coated onto microtiter plates and incubated with purified C3, which was then detected with specific antibodies (**C**). The graphs show the mean and SD of three independent experiments.

To evaluate if aggrecan G3 domain can also activate the alternative pathway, microtiter plates were coated with LCt, Lt and Ct fragments as well as zymosan (positive control for alternative pathway) and BSA (negative control). Then, C3b and C9 deposited from NHS or FB-depleted serum diluted in Mg^++^EGTA, were detected with specific antibodies. Using NHS, C3b (Fig. 6A) but not C9 (Fig. 6B) was found deposited on LCt and Lt domains while zymosan triggered deposition of both complement factors. The C3b deposition was abrogated when using FB-depleted serum (Fig. 6A), which confirms alternative pathway activation by the lectin-containing fragments.

In order to find out how LCt and Lt fragments activate the alternative pathway we investigated if properdin, which can trigger the formation of alternative pathway C3-convertase, would bind to these constructs. However, we only detected binding of purified properdin but not properdin from NHS or secreted from neutrophils (not shown). Thus, the detected interaction with purified protein that has propensity to aggregate [36] could be an artifact not occurring *in vivo* in the presence of all serum proteins.

Since the interaction between G3 fragments and properdin could not be shown convincingly, we tested if any of the other proteins of the alternative pathway could bind the G3 fragments and thereby promote activation. C3 interacted with LCt and Lt to the same extent as to the positive control properdin (Fig. 6C). However, C3 also bound Ct to some extent, in contrast to that Ct could not induce C3b deposition (Fig. 6A). The position of C3 upon binding the different G3 fragments might differ, which could influence how easily C3 is cleaved and activated. We could not detect interactions with FB or factor D (data not shown).

### Aggrecan G3 Domain Binds Complement Inhibitor FH

**Figure 7:**
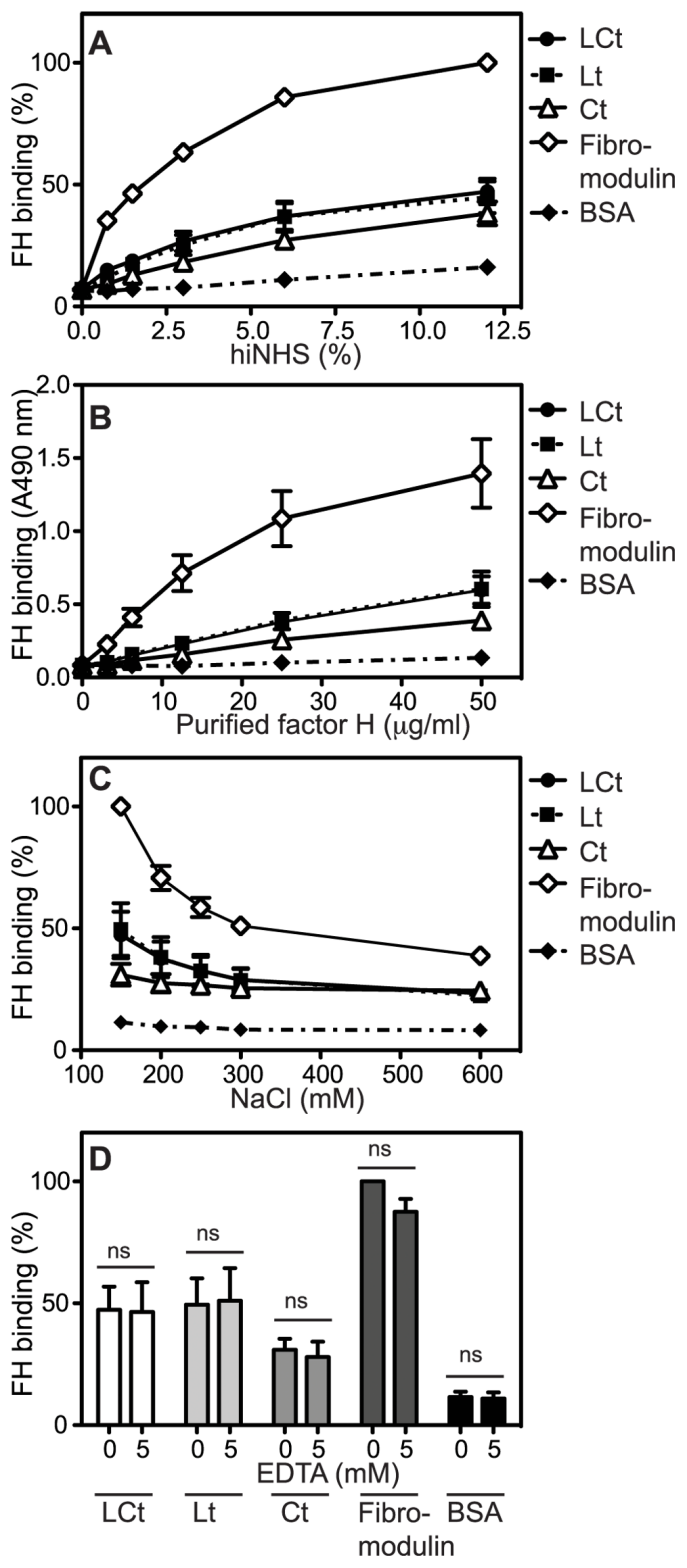
Lectin domain of aggrecan G3 module binds factor H (FH). Microtiter plates were coated with fibromodulin (positive control for binding of FH), LCt, Lt, Ct and BSA (negative control) and the binding of FH from heat-inactivated NHS (hi-NHS) (**A**) or solution containing purified FH (**B**) was detected using specific antibodies. Fibromodulin, LCt, Lt, Ct and BSA were coated onto microtiter plates and incubated with purified FH in a buffer supplemented with increasing NaCl concentrations (**C**) or EDTA (**D**). In A, C and D absorbance was normalized relative to the highest absorbance value obtained with fibromodulin in each figure. The graphs show the mean and SD of three independent experiments. The differences in binding using different EDTA concentrations (D) were not statistically significant, calculated using two-way ANOVA with Bonferroni posttest. (*, p<0.05, **, p<0.01, ***, p<0.001.).

Since there was less C9 deposition than C3b and C4b on LCt and Lt fragments relative to positive control (IgG) both in the classical but mainly alternative pathway activation, we hypothesized that this could be due to binding of complement inhibitors FH and C4BP as previously observed for fibromodulin [6], [37]. We found that FH bound LCt, Lt and Ct, both from hi-NHS (Fig. 7A) and when purified protein was used (Fig. 7B). This indicates a direct interaction between FH and the lectin domain, which is not mediated by deposited C3b. The binding was only partly dependent on ionic interactions, since it was not completely disrupted even in the presence of 600 mM NaCl (Fig. 7C). The interaction was not calcium dependent, as it was not affected by the addition of the chelator EDTA (Fig. 7D). Furthermore, C4BP did not bind G3 domain fragments (not shown). We further investigated the binding sites of FH and C1q to LCt using a competition assay. A mixture of FH in molar excess and C1q was added to coated LCt and the binding of C1q was unaffected by the presence of FH (not shown). Furthermore, C1q in molar excess did not disrupt the binding of FH to LCt either (not shown). This indicates that C1q and FH bind to separate sites on the LCt construct, which has been shown for other endogenous ligands such as fibromodulin [9]. This would allow for simultaneous activation and regulation of complement.

## Discussion

**Figure 8:**
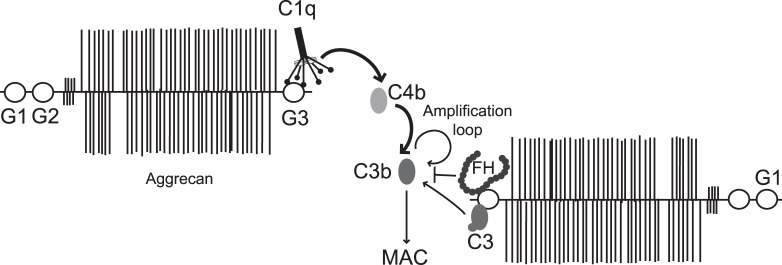
Schematic representation of interactions between aggrecan G3 domain and complement. C1-complex binds the aggrecan G3 domain via the globular heads of C1q, which leads to activation of the classical pathway and the deposition of C4b. Classical pathway C3-convertase is formed, which generates C3b, which is further enhanced by the alternative pathway acting as amplification loop. Furthermore, interaction of G3 with C3 leads to low degree activation of the alternative pathway. Deposited C3b is included in C3- and C5 convertases that finally lead to the formation of MAC. Complement factor FH also binds G3 and is suggested to attenuate the amount of deposited C3b and formed MAC.

A well functioning complement system is important for protection against foreign substances as well as altered self. Imbalance between complement activation and regulation contributes to inflammatory diseases such as RA but also to inflammation in other joint disease, such as OA, particularly at early stages of the disease [26]. In RA, complement can be triggered by immune complexes, apoptotic/necrotic cells and released cartilage proteins and it can further activate parts of both the innate and adaptive immune system. Increasing numbers of inhibitors of complement are being developed for possible use in RA, showing good efficacy in the available animal models of the disease [38], [39]. The first complement inhibitor approved for clinical use, Eculizumab, suppresses complement systemically by inhibiting the cleavage of C5. As a consequence, patients have increased susceptibility for certain infections. It would be more favorable to develop more specific substance acting locally in the joints to limit complement activation. To achieve that, it is vital to increase the understanding of the mechanisms behind cartilage breakdown and complement involvement within the joints. At present, several proteins of cartilage are known to promote inflammation by activating complement. Little is known about the role of aggrecan in the process of complement activation, although generation of MAC in serum incubated with bovine full-length aggrecan was recently reported [26]. In the current study we confirmed that human as well as bovine aggrecan activate complement. More specifically, the ability to activate complement, both via classical and alternative pathway, resides in the CLD of G3 domain of aggrecan (Fig. 8). Interaction site mapping by EM also showed that C1q can bind to central parts of the aggrecan core protein, but we have no data showing whether this particular interaction leads to complement activation. This appears likely since the interaction between both G3 domain and the central core part of aggrecan appear to be mediated by the globular heads of C1q rather than collagenous stalk domains. Such binding to the globular head is typical for known activators of the classical pathway such as IgG and ECM proteins fibromodulin and osteoadherin [6], [9]. Accordingly, interactions of biglycan, decorin and COMP are localized to stalk regions of C1q, which leads to inhibition of the classical pathway presumably due to competition of these ligands with C1r and C1s or steric hindrance preventing the access of ligands (C4 and C2) [5].

Based on the present data one may speculate about the exact binding site for C1q on CLD of G3. CLD binds its ECM ligands in a strictly calcium-dependent manner to the calcium-stabilized loops. Since the binding of C1q to CLD occurs in the presence of EDTA, it indicates separate binding sites of these proteins on CLD. Further, the presence of the EGF or CCP domains in G3 did not affect the C1q binding, which further restricts the possible C1q binding sites on CLD. Nevertheless, the studies of G3 fragments carrying disease-associated missense mutations in the CLD, provide us with additional information about the binding site of C1q. Since the D2267N mutation in CLD partially disturbed the C1q interaction, we suggest that the C1q binding site is in proximity of the mutant amino acid residue, which carries an N-linked carbohydrate probably forming a steric hindrance for C1q-binding. Interestingly, D2267N is associated with spondyloepimetaphyseal dysplasia [22]. However, further efforts will be needed to establish if and how complement may be involved in this disease.

It is plausible that the observed binding of FH to G3 domain is responsible for relatively lower generation of MAC in comparison to activation and deposition of C4b (Fig. 8). We propose that the binding of FH to aggrecan results in inhibition of the alternative pathway C3 and C5 convertases, which in turn yields appreciable decrease in generation of MAC, particularly apparent for the alternative pathway. FH is able to affect later stages of the classical pathway as well, since the alternative pathway, which it controls, acts as amplification loop to the classical pathway. The simultaneous interactions of ligands with C1q and complement inhibitors FH and C4BP are frequently observed for endogenous ligands, for example fibromodulin [6], osteoadherin [6], C-reactive protein [40], amyloid [41], prions [42], DNA [40] and dying cells [43]. This contrasts to the interaction between C1q and immune complexes, in which case no inhibitors bind, resulting in full complement activation. The binding of complement inhibitors to endogenous ligands also interacting with C1q does not completely block complement activation but enables certain level of opsonisation with C3b and its fragments while preventing massive complement activation and assembly of the terminal MAC pathway and release of C5a, which could both trigger significant inflammation. Interestingly, FH interacts not only with CLD but also the construct containing only CCP domain and tail extension. This together with observations that C1q and FH can not compete out each other from binding to the G3 domain and that these two interactions differ in sensitivity to ionic strength, indicate that the binding sites for C1q and FH on G3 are not likely to overlap.

In cartilage, there is a physiological tissue turnover of components with resulting release of protein fragments, including aggrecan, also under normal conditions. In such situation, complement may have an important role in promoting clearance of these fragments to maintain a normal, non-immune stimulatory environment. However, at the time of heavily increased fragment release during RA or OA, excessive complement activation and inadequate regulation could cause a hazardous inflammatory state [44]. What initially disturbs the balance between complement activation and regulation is not clear, but both the availability and concentration of complement triggers in the joint could be crucial factors affecting the detrimental switch in the sensitive balance. It is plausible that aggrecan fragments contribute to the inflammation of the RA or OA joint when released or made more accessible to complement in the partially degraded cartilage. However, this process is most likely not an initial trigger of the disease but acts more as an amplification of the initial activation of the immune system leading to increased proteolytic activity. The nature and amount of released fragments of aggrecan during pathological degradation, could affect their capacity to activate complement and to bind FH.

Aberrant and misguided complement activation is the culprit in many acute and chronic diseases including common joint diseases. In both RA and OA, articular cartilage is destroyed and during this process there is widespread release of cartilage constituents including fragments of aggrecan. We showed that this, in turn, leads to activation of complement providing further rationale for clinical testing in joint diseases of complement inhibitors currently under development.
